# The Importance of Well-Being on Resiliency of Filipino Adults During the COVID-19 Enhanced Community Quarantine: A Necessary Condition Analysis

**DOI:** 10.3389/fpsyg.2021.558930

**Published:** 2021-03-22

**Authors:** Desiderio S. Camitan, Lalaine N. Bajin

**Affiliations:** ^1^College of Arts and Sciences, Manila Tytana Colleges, Pasay, Philippines; ^2^Psycli-Nik Psychological Assessment and Intervention Services, Zamboanga City, Philippines

**Keywords:** positive psychology, well-being, resilience, PERMA, COVID-19, necessary conditions and sufficient conditions for optimality, necessary condition analysis, Philippines

## Abstract

Nation-wide community quarantines and social distancing are part of the new normal because of the global COVID-19 pandemic. Since extensive and prolonged lockdowns are relatively novel experiences, not much is known about the well-being of individuals in such extreme situations. This research effort investigated the relationship between well-being elements and resiliency of 533 Filipino adults who were placed under the nationwide enhanced community quarantine (ECQ) during the COVID-19 pandemic. Participants comprised of 376 females (70.56%) and 157 males (29.45%). The median and mode ages of the participants is 23 years, while 25 is the mean age. PERMA Profiler was used to measure participants’ well-being elements, while Connor-Davidson Resiliency Scale-10 (CD-RISC-10) was used to measure their resiliency. Collected data were analyzed using the regression model and necessary condition analysis. This study corroborated that all the five pillars of well-being are significant positive correlates of resiliency (*p* < 0.00) in quarantined adults. The results shown accomplishment (*β* = 0.447, *p* < 0.01) positively predicts resiliency, while negative emotions (*β* = −0.171, *p* < 0.00) negatively predict resiliency. Lastly, the five pillars of well-being are necessary-but-not-sufficient conditions (ceiling envelopment with free disposal hull, CE-FDH *p* < 0.00) of resiliency. Our results cast a new light on well-being elements as constraints rather than enablers of resiliency. This novel result shows that optimum resiliency is only possible when all the five pillars of well-being are taken care of and when a person is at least minimally contented with their physical health. The present findings underscore the importance of a holistic as against an atomistic approach to maintaining good mental health, which suggests that deficiencies in certain areas of well-being may not be fully addressed by overcompensating on other areas, as all five pillars of well-being are necessary-but-not-sufficient conditions of resiliency. The study ends with the recommendation for the use of necessary condition analysis to study both classical and novel psychological research problems.

## The Importance of Well-Being on Resiliency of Filipino Adults During the COVID-19 Who are Community Quarantined

The infectious Coronavirus disease (COVID-19), which causes respiratory illness includes flu-like symptoms such as cough, fever, and in more severe cases, breathing difficulties. COVID-19 is mainly spread through contact with an infected person who sneezes or coughs. It can be acquired when a person touches their eyes, nose, or mouth after touching objects or surfaces that have the virus on it (World Health Organization, 2020). Starting December 2019, countries imposed travel bans and asked individuals who have possibly been exposed to the contagion to isolate themselves in a dedicated quarantine facility or at home (Brooks et al., 2020) at an unprecedented scale. The Philippines reported its first case of COVID-19 on January 30, 2020. Since then, the number of reported cases exponentially increased by the day (ABS-CBN Investigative and Research Group, 2020). As of December 12, 2020, 447,039 infected cases were reported throughout the country. Of the total number of cases, 409,329 have recovered, and 8,709 have died (Department of Health, 2020).

As a response to the growing threat of the pandemic, the entire Luzon was placed under enhanced community quarantine (ECQ; Medialdea, 2020). Shortly, both Visayas and Mindanao followed suit. The said measure involves draconian restrictions: that include the establishment of checkpoints in most cities; the suspension of classes in all levels; the prohibition of mass gatherings; the temporary shutting down of non-essential businesses; the banning of public utilized utility vehicles; and the strict implementation of home isolation. Although it was initially planned to end on April 12, 2020 (Abueg, 2020), several subsequent recommendations both from the national and local governments extended the nationwide community quarantine until December 31, 2020 (CNN Philippines Staff, 2020). As the nation’s Gross Domestic Product (GDP) shrank 16.5% in the second quarter of 2020, the Philippines officially entered recession as an effect of the extended quarantines (Agence France-Presse, 2020). While quarantine is often among the initial responses against an emerging infectious disease (Parmet and Sinha, 2020), it is often unpleasant for those who are required to submit to it and may lead to several harmful conditions for some persons (Hawryluck et al., 2004; Brooks et al., 2020). Hence, the psychological effects of quarantine have received considerable attention. Barbisch et al. (2015) reported that losing autonomy, isolation away from loved ones, uncertainty, and boredom could lead to adverse effects on an individual’s well-being. Following the imposition of cordon sanitaire in previous outbreaks, substantial anger, anxiety and even an increase in suicide rates have been reported (Brooks et al., 2020). Similarly, the National Center for Mental Health (NCMH) in the Philippines reported that depression and other mental health issues were on the rise after imposing ECQ in different provinces in the country (Tenorio, 2020).

## Well-Being and Its Elements

It is important to note that while quarantines are often unpleasant, their effect on people diverge. While there are individuals who experience mental health issues, there are also those who are more resilient and can move on with their lives. This highlights the importance of studying not only how individuals suffer in light of community quarantines, but also how they cope, and even flourish in the face of such challenging times. Seligman (2011) argued that even in difficult situations, human beings are motivated to thrive and not just merely survive. According to Fredrickson and Losada (2005), flourishing means living “within an optimal range of human functioning, one that connotes goodness, generativity, growth, and resilience.” Based on this definition, resilience appears to arise from flourishing. Well-being predicts resiliency. For clarity, the terms “flourishing,” “thriving,” and “well-being” are used interchangeably in the literature (Butler and Kern, 2016). Therefore, we also use the terms interchangeably here.

Well-being Theory of Seligman (2011) advocates that flourishing arises from five well-being pillars-Positive Emotion, Engagement, Relationships, Meaning, and Accomplishment, hence PERMA. It is important to note that no single element defines well-being, but each contributes to it. Positive emotions include an extensive variety of feelings, which include excitement, satisfaction, pride, and awe. Previous reviews highlight the important role of these emotions in positive life outcomes (Butler and Kern, 2016). Engagement involves activities that stimulate and develop upon an individual’s interests. Csikszentmihalyi (2009) argues that true engagement leads to a state of deep and effortless involvement where an individual is completely absorbed in an activity that often leads to a sense of joy and lucidity. Relationships are social connections important in stimulating positive emotions. They can either be work-related, familial, romantic, and even platonic. The experiences that contribute to well-being are often amplified through our relationships. Positive relationships have been linked to positive outcomes such as better physical health, healthier behaviors, less psychopathology, and lower mortality risk (Tay et al., 2013). A sense of meaning is derived from having a direction in life, belonging to a cause larger than the self, and serving a purpose greater than one’s immediate needs (Steger, 2012). Such activities provide a sense that life is valuable and worthwhile. Various societal institutions such as religion, politics, justice, and community social causes enable a sense of meaning. Accomplishments are pursuits toward and reaching goals, mastery, and efficacy to complete tasks (Butler and Kern, 2016) in various domains such as the workplace, in sports and games, and even in hobbies and interests. Seligman (2011) argued that people pursue accomplishments even when they do not result in positive emotions, meaning, or relationships. Although PERMA was developed mainly within the Western context, several researches found that PERMA is experienced in culturally consistent manners in non-Western societies such as the United Arab Emirates (Lambert and Pasha-Zaidi, 2016), Hong-Kong (Lai et al., 2018), and the Philippines (Nebrida and Dullas, 2018).

## Defining Resilience

Over the past decade, resilience has become a popular concept in both research and clinical practice (Kumpfer, 2002; Walsh-Dilley and Wolford, 2015). Despite the lack of consensus in how it is defined (Vella and Pai, 2019), it is accepted that resilience involves the positive adaptation following a stressful or adverse experience (Porterfield et al., 2010). Most definitions acknowledge two key points about resilience (Herrman et al., 2011). First, is that various factors interact with it. For example, personal characteristics such as personality traits (Oshio et al., 2018), self-esteem (Karatas and Cakar, 2011), and even age (Diehl and Hay, 2010) influence resilience. Social and community factors (Harms et al., 2018) such as secure attachments, the presence of a role model (Levine, 2003), family stability (Grubman, 2018), and culture (Ungar, 2008) affect the ability to cope with daily struggles. Second, resilience is time and context-specific and may not be present across all life domains. Resilience appears to be receptive to the influence of specific situations (Hayman et al., 2017) such as unique stressors (Jex et al., 2013) like war and other happenstances (Besser et al., 2014).

While the aforementioned literature provides key insights into the definition, factors, and contexts of resilience, most research focuses on factors are outside the control of the individual. While these researches are important in explaining the development of resilience, they lack emphasis on positive mechanisms, which are behaviors a person can perform to facilitate resilience. While resilience has been studied both in daily and unique stressors, none focused on the novel situation of wide range community quarantines. Therefore, despite the abundance of resilience-related research, the question remains “What positive mechanisms are involved in the resilience of people who are subjected to quarantine?”

## The Present Study

In this paper, we introduced a novel approach in understanding the necessary but not sufficient nature of the aforementioned positive aspects of well-being in predicting resiliency. We used Dul (2016) Necessary Condition Analysis (NCA), which seeks to identify necessary-but-not-sufficient conditions in data sets (Dul, 2018). A necessary condition is a crucial factor in an outcome. If it is not in place, the outcome will not be achieved, but its sole presence does not guarantee that the outcome will be obtained. Without the necessary condition, however, there is a certain failure, which may not be compensated by other determinants of the outcome. Necessary (but not sufficient) conditions widely exist in real-life. For example, the novel SARS-CoV-2 coronavirus is a necessary-but-not-sufficient condition for COVID-19 (World Health Organization, 2020). Without SARS-CoV-2 coronavirus, an individual will not acquire COVID-19. However, even with SARS-CoV-2 coronavirus, an individual may or may not acquire COVID-19. In the same light, a college student who wants to pass the course, Introduction to Psychology (the outcome) needs to attend 80% of lecture hours (necessary conditions). However, attending class regularly does not guarantee passing the course as other requirements (examinations, seat-works, research work, and journal critique paper) play a role in a student’s grade. Yet, if the student incurs too many absences and tardiness, failure is guaranteed. As seen in the aforementioned examples, necessary causes are not automatically sufficient. They can be seen as constraints, barriers, or obstacles one needs to deal with to arrive at the desired outcome.

While well-being and resiliency are closely related concepts (Hu et al., 2015) *Flourishing* model of Seligman’s (2011) perceives resiliency as the result of both “surviving” and “thriving” psychological characteristics. This theoretical relationship between well-being and resilience has gained empirical support in recent years (Harms et al., 2018). For example, Martínez-Martí and Ruch (2017) and Burns and Anstey (2010) demonstrated that measures of well-being are not simply redundant with self-report scales of resilience. At the same time, while the relationship between these two concepts are robust, it is rarely straightforward (Harms et al., 2018). Interestingly, some researchers (Fredrickson et al., 2003; Tugade and Fredrickson, 2004; Ong et al., 2006, 2010; Kuntz et al., 2016) argued that optimal levels of PERMA elements predict resilience in normal sample.

In the light of the foregoing, the present study aims to investigate how PERMA predicts the resiliency of community quarantined individuals. An explanation of possible necessary-but-not-sufficient conditions of resiliency during quarantine may have both theoretical and practical value. Theoretically, an investigation of this sort allows the advancement of our understanding of how a multitude of variables coalesces to produce resiliency in times of quarantine and social isolation. This is significant as wide-range and prolonged quarantines are relatively novel experiences. Hence, not much is known about its psychological implications for human beings. Psychological interventions may target different necessary-but-not-sufficient variables jointly. Because of NCA’s ability to identify *bottleneck* variables (Dul, 2019a), conditions that must be present for resiliency to be possible, interventions may prioritize bottleneck variables of resiliency to maximize the use of limited resources. Lastly, identifying necessary-but-not-sufficient conditions for resiliency may also help individuals who are quarantined to develop their understanding of the behaviors they need to engage to have resiliency. Following this logic, we hypothesize that:

*H_01_:* PERMA elements predict the resiliency of the community-quarantined individuals.
*H_02_:* PERMA elements are necessary, but not sufficient conditions, for the resiliency of the community-quarantined individuals.

## Methodology

### Research Design

To test the assumption that PERMA elements are both sufficient and necessary conditions of resiliency in community quarantined individuals, sufficiency and necessity observational design were used concurrently. In these designs, the conditions (PERMA) and the outcome (resiliency) are observed in real-life context and without the manipulation of the condition. While sufficiency and necessity observational research designs follow the same data gathering procedures, they diverge in data analysis. Dul (2016) argued that NCA is a complement to traditional approaches to analyze relations. As in our research, by using multiple regression we could spot determinants that contribute to resiliency, whereas NCA allowed us to spot critical determinants (constraints) that prevent resiliency from developing. These bottlenecks, when present, prevents resiliency from occurring even when we increase the values of other determinants unless we take away the bottlenecks by increasing the value of the critical determinant. NCA lead us to discover critical determinants that were not part of the determinants identified with the regression model. Using both approaches is critical in adequately understanding the resiliency of individuals who are subjected to the extended ECQ.

### Research Participants

Because of the restrictions in both mobility and social interactions as direct consequences of the nationwide ECQ, we used purposive – convenience sampling to recruit Filipino Facebook users who reside in communities placed under the ECQ. The survey was promoted through social media, primarily on Facebook. A total of 541 participants responded to our online survey *via* Google Form. The minimum age reported was 16 years old, while the maximum age was 64 years old with a median of 23. Because resiliency scores are contingent to age, only those whose ages ranged between emerging adulthood to early middle adulthood (18–40) were included in the study.

#### Inclusion Criteria

Participants that were considered to partake in the research met the following criteria: first, a participant must be aged 18 to 40 years old. Second, he/she resides in a quarantined area in the Philippines. Third, a participant must be a Filipino citizen as social and cultural factors influence resiliency.

#### Exclusion Criteria

A participant was excluded in the research because of the following conditions: first, a participant aged less than 18 years old and over 40 years old, a participant who refused to completely answer the online survey questionnaires, and a participant who does not reside in a quarantine area in the Philippines.

#### Ethical Considerations

In dealing with the participants, respect and protection of the privacy of the participants were prioritized. Thus, privacy and anonymity was of paramount importance. Also, voluntary participation of the chosen participants for said the study was important. Participants had the right to withdraw from the study at any phase of the research if they wished to do so.

Potential participants were fully informed regarding the research, full consent was essential and obtained from the participants. The first page of the online questionnaire required participants to check a box to show consent before having access to the survey. The principle of informed consent involved the researchers providing sufficient information and assurances about taking part to allow potential participants to understand the implications of participation and to reach a fully informed, considered, and freely decided about whether to do so, without the exercise of any pressure or coercion. No incentives were provided in return for their participation.

In collecting data through online surveys, we minimized intrusions on privacy, anonymity, and confidentiality. Before data collection, an adequate level of confidentiality of the research data was ensured to the participants to make them feel secured and protected with the information they shared or contributed. Also, any communication about the research was observed with respect and transparency. Ultimately, research participants are not subjected to harm.

### Research Instruments

Google Forms was used to gather sociodemographic variables from the sample and deliver the following self-administered scales, which were used to measure the variables of the current study. Specifically, we used the Connor-Davidson Resiliency Scale-10 (CD-RISC-10) to measure their resiliency, and the PERMA Profiler to measure participants’ well-being elements.

### Connor-Davidson Resiliency Scale

The CD-RISC-10 is a 10 item scale that is used to measure resiliency, operationally defined as the ability to “thrive in the face of adversity” (Connor and Davidson, 2003). The unidimensional CD-RISC-10 evaluates several components of psychological pliability: the abilities to adapt to change, manage what comes along, handle stress, stay focused and think clearly, avoid getting discouraged in the face of failure, and handle unpleasant emotions such as pain, sadness, and anger (Campbell-Sills and Stein, 2007). Each item is rated on a five-point range of responses. The total score is computed by getting the sum of all the responses whereby higher scores show high resilience (Scali et al., 2012). Campbell-Sills et al. (2009) maintained that CD-RISC-10 has a median score of 32 with lowest to highest quartiles of 0–29 (Q1), 30–32 (Q2), 33–36 (Q3), and 37–40 (Q4) in general sample. As a widely used scale, CD-RISC-10 has achieved remarkable internal consistency of 0.89 in general population samples. It is both valid and reliable within the context of different cultures, including Filipino samples (Campbell-Sills and Stein, 2007).

### PERMA Profiler

The PERMA Profiler is a brief scale that measures the five pillars of well-being: positive emotion, engagement, positive relationships, meaning, and accomplishment, together with negative emotions and health (Butler and Kern, 2016) along a 10-point Likert type scale. Of the 23 items, 15 correspond to the five core elements of well-being (three items per PERMA domain). In addition, eight items were included to test negative emotions (three items), physical health (three items), loneliness (one item), and overall well-being (one item). All items are expressed positively and higher scores denote better well-being except for negative emotions. Subscale scores are calculated by getting the mean of the three items on each subscale, except for loneliness. Overall well-being is calculated by averaging all items except those from the negative emotions subscale. The measure has been used in various samples and was found to have sufficient psychometric properties (Cobo-Rendón et al., 2020). Butler and Kern (2016) reported that adequate reliability is observed for overall well-being and all subscales, *α* range from 0.71 to 0.94 across eight studies (*N* = 31,966). According to Nebrida and Dullas (2018), the Tagalog version of the PERMA Profiler has a Cronbach’s alpha of 0.842 in 101 Filipino participants.

In the current study (*n* = 533), both PERMA Profiler (*α* = 0.927) and CD-RISC-10 (*α* = 0.915) have an “excellent” internal consistency. These results confirm that the scales are reliable tools for measuring elements of Well-being and Resiliency, respectively, in our sample.

### Data Gathering Procedures

Data gathering lasted from March 23 to April 10, 2020, during the first reset of the nationwide extended ECQ. After securing individuals’ interest to take part in the study, we sent potential participants a link to the survey *via* Facebook Messenger. The first section of the Google Form shows the title of the research and an overview of the current study. After giving consent, participants could fill out the survey. Participants cannot answer the scales without explicitly agreeing to partake in the study. After securing informed consent, each participant was asked to provide their sociodemographic characteristics and then answer the PERMA Profiler and the CD-RSC-10. Answering both scales did not take the participants more than 20 min. After completing the questionnaire, each participant was virtually debriefed.

At any point, should a participant decide not to proceed with the research, they were free to do so with no implications. All the participant has to do was to close the Google Form window and any previously provided data were not recorded.

Data from Google Form were exported to IBM’s Statistical Package for Social Sciences (SPSS) and NCA Software for data analysis.

### Data Analysis

Frequency and percentage were used to analyze the sociodemographic characteristics of the participants. We used Cronbach’s alpha to determine the internal reliability of the measuring scales. Correlation and multiple regression analyses were conducted to examine the relationship between PERMA elements and potential predictors of resiliency. Lastly, we used NCA to analyze whether the core elements of well-being are necessary but not sufficient conditions of resiliency.

There are two steps in NCA (Dul et al., 2019), determining ceiling lines and bottleneck tables are the first. Unlike traditional regression models where a line is drawn through the middle of the data in an XY-plot, a ceiling line is created in NCA. This line distinguishes between areas with cases and areas without cases, the zone found in the upper left-hand corner of the plot. However, exceptions such as outliers and errors may be present in a sample so that the empty zone above the ceiling is not empty (Karwowski et al., 2016). The ceiling line is a non-decreasing line (either a linear step function or a straight line) that shows which level of *x* (well-being elements) is necessary but not sufficient in producing the desired level of *y* (resiliency).

Dul (2016) identified two techniques in drawing the ceiling line. The first is the non-parametric Ceiling Envelopment with Free Disposal Hull (CE-FDH), which is a piecewise linear line. It is the default ceiling envelopment technique for NCA because it is flexible and intuitive and applies to dichotomous, discrete, and continuous conditions. The second technique is the parametric Ceiling Regression with Free Disposal Hull (CR-FDH), unlike the CE-FDH, this technique smoothens the piecewise linear lines by using a straight line. Because of this, CR-FDH usually has some observations above the ceiling line. Whereas CE-FDH does not. In further comparing the two techniques, CE-FDH is preferred when a straight line does not represent the data because smoothing reduces the size of the ceiling zone as with dichotomous variables and for discrete and continuous variables with relatively low small data sets. CE-FDH is 100% accurate in drawing the demarcation between observations above and observations below the ceiling line.

Quantifying the accuracy of ceiling lines, effect size, and statistical significance of the necessary conditions and necessary inefficiency are the second and final step (Dul et al., 2020). The area of the empty zone above the ceiling line divided by the area where cases would be possible given the minimum and maximum values of X and Y is the effect size of a necessary condition (Karwowski et al., 2016). Therefore, large effect size shows lower ceiling line and greater limitations that well-being elements have on resiliency. On the other hand, if there is a lack of empty space in the scatter plot then well-being elements are not contingents of resiliency. The effect size of a necessary condition can take the values between 0 and 1 where 0–0.1 corresponds to a small effect, 0.1–0.3 a medium effect, 0.3–0.5 a large effect, and *d* that is greater than 0.5 a very large effect (Tynan et al., 2020). An R package that allows the calculation of various effect size indicators and inferential statistics useful for hypothesis testing is provided by Dul (2016). The NCA null hypothesis is that the observed effect size is the same as the effect size calculated using random data (Dul, 2019b). An estimation of the probability that the observed necessary condition effect size results from comparing two unrelated variables, otherwise known as permutation test, is used to determine statistical significance in NCA (Dul et al., 2020). Observed values of the *x* and *y* variables are randomly paired without replacement. Such pairing continues until the sample size is reached and the process is repeated at least 10,000. The resultant value of *p* is interpreted using traditional thresholds such as *α* = 0.05 or *α* = 0.01. Depending on the context of the research, both significance testing and effect size are useful in determining the theoretical and practical importance of an observed outcome (Tynan et al., 2020). We focus our attention on conditions with both *d* > 0.5 and *p* < 0.05.

SPSS was used to analyze the frequency and percentage of various sociodemographic variables, the scales’ reliability, and for generating the Regression Model. R Statistical Software with NCA Package was used to conduct NCA.

## Results

### Profile of the Participants

Participants comprised 376 females (70.56%) and 157 males (29.45%). The median and mode ages of the participants are 23 years, while the mean age is 25. Among the participants 189 (35.46%) were college students, 293 (54.97%) are employed, and 51 (9.57%) are out of work. Lastly, seven (1.31%) participants reported that they had direct contact with someone who was infected with COVID-19, while 100 (18.76%) reside in communities with known COVID-19 cases and 426 (79.92%) have no exposure to the disease.

### PERMA as Predictors of Resiliency

Table 1 summarizes the descriptive statistics and analysis results of the study. Results revealed that the mean resiliency score of the participants is 24.83, with a SD of 7.22. PERMA elements including overall well-being are positive and significantly correlated with resiliency. Interestingly, a subjective sense of health (feeling good and healthy each day) showed only a weak, albeit significant positive correlation with resiliency. Negative emotions and loneliness are negatively correlated with resiliency.

**Table 1:** Summary statistics, correlations, and coefficient results for regression analysis of study variables.

Variables	Mean	SD	*R*	R*p*	*β*	*B*	*p*
Resiliency	24.83	7.22					
Positive Emotions	7.13	2.03	0.54	0.00	0.271	0.963	0.25
Engagement	7.36	1.85	0.40	0.00	0.142	0.556	0.44
Positive Relations	7.31	2.06	0.46	0.00	0.126	0.440	0.57
Meaning	7.27	2.10	0.53	0.00	0.239	0.820	0.29
Accomplishment	7.04	1.86	0.55	0.00	0.447	1.85	0.01
Overall Well-being	7.27	1.55	0.57	0.00	−0.583	−2.72	0.54
Health	7.41	1.58	0.261	0.00	0.143	0.66	0.42
Negative Emotions	5.62	2.17	−0.03	0.516	−0.171	−0.57	0.00
Loneliness	5.23	2.87	−0.07	0.96	−0.028	0.573	0.57

R, Pearson correlation coefficient with resiliency; R*p*, *p* value of R; Loneliness and health *R* = −0.211, where *p* = 0.001. *β*, standardized beta; *B*, unstandardized beta; *p*, probability value of PERMA elements as predictors of resiliency. *R*^2^ of five original PERMA elements including four additional subscales = 0.368.

The multiple regression model with all nine predictors produced *R*^2^ = 0.368, *F*(9, 523) = 33.83, *p* < 0.001 with adjusted *R*^2^ = 0.357. This means that 36.8% of the variance in resiliency scores is because of the PERMA elements. As seen in Table 1, accomplishment (*β* = 0.447, *p* < 0.01) and negative emotions (*β* = −0.171, *p* < 0.00) are the only elements of PERMA with significant regression weights, showing scores on these elements predict resiliency. However, negative emotions have significant negative weight as compared to with standardized coefficients of −0.171 vs. 0.477.

The multiple regression model of the four confounders between the relationship of PERMA elements and resiliency produced *R*^2^ = 0.036, *F*(4, 528) = 4.90, *p* < 0.001 with adjusted *R*^2^ = 0.028. It shows that the spread of the confounders is 3.6% between the relationship of the variables. As seen in Table 2, only employment status (student, unemployed, and employed) with *β* = 0.14, *p* < 0.00 is a significant predictor of resiliency.

**Table 2:** Confounders between the relationship of PERMA and Resiliency.

Variables	*R*	*β*	*B*	*p*
Resiliency				
Gender	0.01	0.02	0.24	0.73
Age	0.14	0.08	0.09	0.08
Exposure to COVID-19	0.01	0.00	0.06	0.93
Employment status	0.17	0.14	1.09	0.00

*R*, Pearson correlation coefficient with resiliency; *β*, standardized beta; *B*, unstandardized beta; *p*, probability value of confounders with *p* < 0.001.

#### PERMA as Necessary-But-Not-Sufficient Conditions of Resiliency

**Figure 1:**
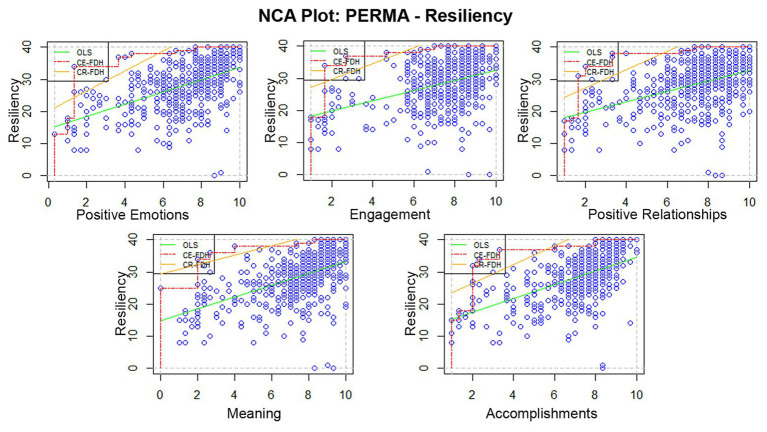
Scatterplots of the original PERMA elements (*x*) as necessary conditions of resiliency (*y*). Note: The dashed lines are ceiling lines. The selected ceiling line technique (CE-FDH) do not allow data points above the ceiling line. The solid line is the ordinary least squares regression line.

The results of NCA on Resiliency show that all five elements of the original Seligman (2011) PERMA are necessary but not sufficient conditions of Resiliency among individuals who are community quarantined as showed by the size of the empty zone in the XY-plots in Figure 1. This means that to score 35 in the CD-RISC-10, a score of 1 for positive emotions and engagement, a score of 2 for Positive Relationships, Meaning, and Accomplishment are necessary.

**Figure 2:**
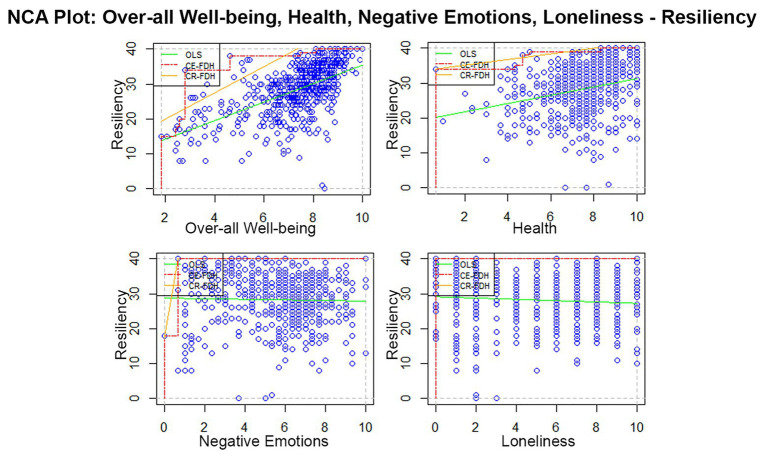
Scatterplots of overall well-being, health, negative emotions, and loneliness (*x*) as necessary conditions of resiliency (*y*). These elements were not in the original Seligman (2011) PERMA model but are supplementary subscales in Butler and Kern (2016) PERMA Profiler. Note: The dashed lines are ceiling lines. The selected ceiling line technique (CE-FDH) does not allow data points above the ceiling line. The solid line is the ordinary least squares regression line.

Figure 2 contains the scatterplots of the four supplementary subscales of Butler and Kern (2016) PERMA Profiler. Only the xy-plot of Overall Well-being (*x*) and Resiliency (*y*) has a “moderately sized” empty zone in the upper left corner of the plot. This is not surprising considering that Overall Well-being is the composite score of the five PERMA elements and health score. The scatterplots of Health (*x*) and Resiliency (*y*), and Negative Emotions (*x*) and Resiliency (*y*) contain discernibly small empty zones. Lastly, the empty zone is absent in the Loneliness (*x*) – Resiliency (*y*) scatterplot. This assumes that Loneliness is not a necessary condition of Resiliency as the presence and size of an empty zone is a sign that a necessary condition is present (Dul, 2016).

We summarized the results of the multiple NCA in Table 3. The observed accuracy of all variables exceeds arbitrary benchmark of Dul (2018) for the desired accuracy of 95%. Dul suggests the use of CR-FDH for interpreting variables with accuracies above 95%. However, since our variables do not follow a normal distribution (*p* = 0.00) based on One-Sample Kolmogorov-Smirnov Test, we used the non-parametric CE-FDH ceiling line technique. Necessary-but-not-sufficient relationships between Resiliency and the five original PERMA elements and the auxiliary components are observed. The NCA effect size range between *d* = 0.09 and 0.12 based on CE-FDH for the original PERMA elements and *d* = 0.04 to 0.12 on the supplementary elements, excluding Loneliness. According to recommendations, Positive Emotions, Meaning, Accomplishment, and Overall Well-being of Dul (2016) have medium effect sizes on Resiliency. Engagement, Positive Relationships, Negative Emotions, and Health have small effect sizes on Resiliency. The NCA significance test is powerful enough to rule out an effect being the product of randomness (Dul et al., 2020). Lastly, there is no necessary-but-not-sufficient relationship between Loneliness and Resiliency.

**Table 3:** Necessary conditions effect size and significance test for PERMA Profiler subscales predicting Connor-Davidson Resiliency Scale-10 (CD-RISC-10) scores.

	CE-FDH	CE-FDHp	CR-FDH	CR-FDHp	Accuracy (%)	Skewness	Skewness p
Positive Emotions	0.12	0.001	0.15	0.001	98.5	−1.18	0.00
Engagement	0.09	0.001	0.09	0.001	99.4	−1.56	0.00
Positive Relations	0.09	0.001	0.12	0.001	98.9	−1.19	0.00
Meaning	0.12	0.008	0.10	0.008	99.4	−1.21	0.00
Accomplishment	0.12	0.001	0.13	0.001	98.7	−1.37	0.00
Overall Well-being	0.12	0.001	0.17	0.001	97.7	−1.32	0.00
Health	0.07	0.21	0.06	0.27	99.4	−0.45	0.00
Negative Emotions	0.04	0.23	0.02	0.52	100	−0.81	0.00
Loneliness	0.00	1.00	0.00	1.00	100	−0.23	0.00

CE-FDH, ceiling envelopment with free disposal hull; CR-FDH, ceiling regression with free disposal hull. The *p* value reported was estimated with 10,000 permutations and are treated as significant if <0.05. The threshold for statistical significance is arbitrary but commensurate with the example given by Dul et al. (2020). Accuracy refers to the percentage of observations under the CR-FDH ceiling line. Skewness *p* is based on One-Sample Kolmogorov-Smirnov Test. Skewness of resiliency scores is −0.78.

The ability to identify bottleneck variables (constraints) is a useful feature of NCA, especially for interpreting multivariate necessary conditions (Dul, 2019b). Table 4, which is read horizontally, shows for which level of resiliency, which level of PERMA elements is necessary. For a desired value of resiliency, in the first column, it shows the minimum required values of the PERMA elements in the next columns. Levels are expressed in percentage ranges so that 0 is the minimum value, the maximum is 100, and 50 is the point between these two values.

**Table 4:** Bottleneck table of PERMA elements as necessary conditions of resiliency based on CE-FDH.

Re	P	E	R	M	A	OW	H	N	L
0	NN	NN	NN	NN	NN	NN	NN	NN	NN
10	NN	NN	NN	NN	NN	NN	NN	NN	NN
20	NN	NN	NN	NN	NN	NN	NN	NN	NN
30	NN	NN	NN	NN	NN	NN	NN	NN	NN
40	6.9	NN	NN	NN	3.7	7.1	NN	NN	NN
50	10.3	7.4	7.4	NN	11.1	9.1	6.7	NN	NN
60	10.3	7.4	7.4	NN	11.1	11.6	6.7	NN	NN
70	10.3	7.4	7.4	20.0	11.1	11.6	6.7	NN	NN
80	10.3	7.4	11.1	20.0	11.1	11.6	6.7	NN	NN
90	34.5	18.6	25.9	26.7	25.9	34.2	6.7	42.9	NN
100	75.9	66.7	70.3	83.3	77.8	76.8	6.7	82.1	NN

Re, resiliency; P, positive emotions, E, engagement; R, positive relationships; M, meaning; A, accomplishment; OW, overall well-being; H, health; N, negative emotions; L, loneliness; NN, not necessary.

The bottleneck table shows that no minimum value of any PERMA element is necessary to score 30% in Resiliency. This means that at 30% no PERMA element is a bottleneck for resiliency. However, for a resiliency level of 40%, the minimum required level of Positive Emotions is 6.9%, the necessary level of Accomplishment is 3.7, 7.1% for Overall Well-being, and none of the over PERMA elements are necessary. As observed in the bottleneck table, when Resiliency increases from 0 to 100%, more PERMA elements become necessary, and required levels of the PERMA elements become higher. At 90% level of Resiliency, the necessary level of Positive Emotions is 34.5%, Engagement is 18.6%, Positive Relationships is 25.9%, Meaning is 26.7%, Accomplishment is 25.9%, Overall Well-being is 34.2%, Health is 6.7%, and Negative Emotions is 42.9%. No level of Loneliness is necessary for any level of Resiliency. Not achieving any of these minimum levels means that attaining a 90% level in resiliency is impossible. Since each condition is a bottleneck, scoring higher in other elements does not compensate for the deficiency in others.

## Discussion

Wide range community quarantines and social distancing are elements that are increasingly becoming the new normal as a result of the global COVID-19 pandemic. Previous research (Hawryluck et al., 2004; Barbisch et al., 2015; Brooks et al., 2020; Parmet and Sinha, 2020) offer invaluable insights into the psychological consequences of restrictions. Moreover, while there has been an interest in the psychological impact of COVID-19 and community quarantine in the Philippines (for example, Nicomedes and Avila, 2020; Tee et al., 2020), most focus on the negative psychological impact of COVID-19. This raises the question of what protective factors are important in the midst of prolonged community quarantines. To test this properly, we used a combination of the traditional regression model and the novel multivariate necessary-but-not-sufficient conditions analysis to investigate how resiliency is contingent on well-being elements in Filipinos who are community quarantined.

Participants of this study were predominantly female, around the age of 23 and who are employed. While, we specifically targeted individuals between the ages of 18–40, most of our sample are emergent adults (mean age = 25, median, and mode ages = 23). The disproportional representation of young adult females can be attributed to several factors. First, previous studies (Smith, 2008; Yetter and Capaccioli, 2010; Slauson-Blevins and Johnson, 2016) have reported that young adult females take part in online surveys at a higher frequency compared with their male counterparts. There are more female Facebook users than males (Lee et al., 2016), which is significant because we invited potential participants through Facebook. Lastly, the Philippines has a young population. The median age in the Philippines is 25.7 (United Nations Statistics Division, 2019; Plecher, 2020). Taken together, it can be assumed that the sociodemographic characteristics of our study are similar to the Filipino Facebook population.

Based on the CD-RISC-10 quartiles for community sample provided by Campbell-Sills et al. (2009), the mean resilience score (24.83) of the current sample belongs to the lowest 25%. This implies that the participants of the current study have lower resiliency scores than the general population. This result ties well with the notion that resilience is stress-context specific (Jex et al., 2013; Wood and Bhatnagar, 2015; Hayman et al., 2017) and that the nature of the sample influences resiliency scores (Connor and Davidson, 2003). Specifically, people with psychiatric problems and those who are experiencing significant stress score lower than the general population (Li et al., 2012; Ye et al., 2017). In the context of COVID-19, Nicomedes and Avila (2020) found that Filipinos in community quarantine experience significant stress and scored high on both health anxiety and panic.

While resiliency and well-being have become commonplace terms and construct central in positive psychology (Jeste et al., 2015), they are often studied using correlational methods (Schultze-Lutter et al., 2016), and traditional approaches *via* the sufficiency paradigm. In line with previous studies (Souri and Hasanirad, 2011; Khawaja et al., 2017; van Agteren et al., 2018), we found that all elements of well-being are positively correlated with resiliency. Although the multiple regression test shows that among the original PERMA elements, only accomplishment is a significant predictor of resilience. This means that the subjective sense of competence, having a structure each day, i.e., identifying, setting, and achieving daily goals enable resiliency in individuals subjected to quarantine. We also observed that negative emotions significantly, although negatively predict resilience. This suggests the significant predicting function of individuals’ tendency to experience anxiety and anger for lower levels of resilience. These findings support the previously reported (Tugade and Fredrickson, 2004; Chen et al., 2018) link between negative emotions and low levels of resilience.

In this paper, we identified elements of well-being that are necessary-but-not-sufficient for resiliency to occur in individuals who are community quarantined. Specifically, Positive Emotions, Meaning, and Accomplishment are significant and moderately necessary conditions of Resiliency, as suggested by their medium effect size. This finding suggests that positive feelings like interest, joy, and contentment and pursuing a daily purpose, and regularly experiencing a sense of accomplishment are essential to quarantined individuals’ ability to thrive in their present predicament. Such necessary conditions not only allow individuals to enjoy everyday experiences (Abiola et al., 2017) but also provide a sense that life matter, which replenishes depleted energy from adverse experiences, and are required in the development of resiliency.

Engagement and Positive Relationships have small yet significant effect sizes on Resiliency. This infers that experiencing a state of “flow,” or being absorbed in an activity (Nakamura and Csikszentmihalyi, 2014) and feeling loved, supported, and valued by others are also necessary to the quarantined individuals’ capacity to recover quickly from their daily difficulties. This ties well with previous studies (Eaude, 2009; Svence et al., 2015; Abiola et al., 2017; Gerino et al., 2017; Roncaglia, 2017; Cobo-Rendón et al., 2020), where well-being elements were observed to be related with the occurrence of resiliency in individuals from a different context. Well-being elements allow quarantined individuals to focus their attention on alleviating harm, preventing negative mental health consequences, and finding positive outcomes in the presence of difficulty.

A unique finding, we encountered is that PERMA elements are bottleneck variables of resiliency. This highlights the little-known capacity of well-being to serve as a constraint to attaining higher levels of resiliency in community-quarantined individuals. This novel result shows two things. First, low levels of resiliency (30% and less) do not necessitate even the slightest well-being elements. Second, higher levels of resiliency require certain levels of all the original PERMA elements and physical health. However, health remains a constant, albeit weak, necessary condition. This means that optimum resiliency is only possible when all the five pillars of well-being are taken care of and when one is at least minimally content with their physical health. When comparing our results to those of older studies (Sanders et al., 2015; Svence et al., 2015; Abiola et al., 2017). It must be pointed out that while the link between well-being and resiliency has been suggested in these studies, none could establish the necessary-but-not-sufficient relationship between the concepts. The present findings underpin the importance of holistic rather than an atomistic approach to mental health as noted by Mario (2012) and contradicts the compensation hypothesis of well-being. NCA revealed that deficiencies in certain areas of well-being may not be addressed by overcompensating in other areas, as all five pillars of well-being are necessary-but-not-sufficient conditions of resiliency.

Our findings show that loneliness is inversely correlated with the subjective perception of health. This basic result is consistent with the research (Balter et al., 2019) showing that loneliness predicts poor immune systems in healthy young adults. This is important since maintaining good health is vital amidst a growing viral pandemic. We observed that loneliness is a significant negative predictor of resiliency and not a necessary condition for any level of resiliency in individuals who are community quarantined. A similar conclusion was reached by Perron et al. (2014) where individuals who feel resilient also experience less loneliness. This further highlights the importance of the elements of well-being as necessary conditions of resiliency, which may lessen the effects of or serve as a buffer against loneliness and other negative psychological consequences of quarantine.

The overall results of our study have theoretical and practical implications. At a theoretical level, our results found clear support to PERMA concept of Seligman (2011) as necessary ingredients of resiliency even for socially isolated individuals such as those placed in ECQ. This goes beyond previous reports wherein PERMA elements were observed as predictors of resiliency, as only NCA can identify a necessary-but-not-sufficient relationship between the said variables. Despite experiencing segregation like lockdowns, the conditions that will allow people to thrive in the face of adversity are the same as when they are not undergoing such a predicament. Therefore, this finding can help us understand how the five elements of well-being constrain the negative psychological consequences of community quarantine by providing a buffer against these harms, reducing their effects, and promoting individual capacity to cope with such unsettling conditions. From this standpoint, we speculate that PERMA should be inversely correlated with negative indicators of mental health and correspondingly with other elements of positive psychological health, as noted by Hu et al. (2015). At a practical level, this opens an opportunity to develop evidence-based interventions such as telepsychology (Zhou et al., 2020) for quarantined individuals that help clients understand behaviors they need to engage to have resiliency, and target multiple necessary-but-not-sufficient variables jointly, and not just focus on certain elements of well-being. This provides support for eclectic approaches to therapy especially the ones that incorporate positive psychology as Bolier et al. (2013) noted empirical support for the effectiveness of such interventions. Lastly, our findings agree with the call to a more inclusive psychology in the Philippines. This paradigm shift involves incorporating such approaches as critical (Paredes-Canilao et al., 2015) and positive (Datu et al., 2018) psychology to the prevailing traditional pathology-based perspective.

One fundamental limitation of this study is that the use of multiple regression and NCA cannot guarantee causality (Dul, 2016). While our data is consistent with the causal hypothesis, it is not evidence of a causal connection. Therefore, causal necessary-but-not-sufficient relations should not be inferred from our data. Another important caveat in interpreting our results is that we used the Facebook population as compared to the actual geographical population. It is not a perfect representative since Facebook users are usually younger females who have better educational attainment compared to the general population (Kosinski et al., 2015). Resiliency and well-being were measured during the ECQ, a far from normal situation. Therefore, although we took obligatory safety measures to increase the trustworthiness of the findings, we suggest that care be exercised when generalizing our findings into the general population and normal circumstances.

Many questions remain to be answered concerning the well-being of people who are community quarantined and the utility of NCA in psychological research. Further work is needed to identify the negative consequences of prolonged quarantine on individuals, especially those who have preexisting mental health problems and those who experience a disruption in access to their mental health-care providers. Moreover Odacı and Kalkan (2010) reported that internet use, specifically social media (Maglunog and Dy, 2019) exacerbates loneliness and that social media usage is expected to rise during the ECQ. Another important question, therefore, is how does the ongoing quarantine affects rates and levels of loneliness. Finally, while necessary conditions are traditionally studied using regression analysis in psychological research, NCA proved to be a more useful tool in understanding necessary-but-not-sufficient relationships because of its ability to understand bottleneck variables. We, therefore, recommend the use of NCA in both classical and novel psychological research problems.

Resiliency grants us the capacity to flourish in the face of difficulty. For resiliency to result, the pillars of well-being are essential. Our research reveals, however, that well-being elements could be enablers or constraints. Accomplishment, for example, could predict resiliency. All pillars are necessary to attain it. Compensating in certain aspects cannot address the deficiency in others. Herein lies the importance and significance of holistic well-being. Those who can attain this are better equipped to thrive in the ECQ, a situation that affects the lives of so many Filipinos.

## Data Availability Statement

The original contributions presented in the study are included in the article/Supplementary Material, further inquiries can be directed to the corresponding author.

## Ethics Statement

The studies involving human participants were reviewed and approved by Manila Tytana Colleges Research Ethics Committee. The patients/participants provided their written informed consent to participate in this study.

## Author Contributions

DC wrote the introduction, results, and discussion and conducted the necessary condition analysis. LB wrote the methods, contributed in the results and discussion, and conducted the correlation analysis, regression analysis, and reliability check of the scales. All authors contributed to the article and approved the submitted version.

### Conflict of Interest

The authors declare that the research was conducted in the absence of any commercial or financial relationships that could be construed as a potential conflict of interest.
